# O033: Is MRSA inpatient transmission driving high MRSA hospital importation in the US veterans affairs?

**DOI:** 10.1186/2047-2994-2-S1-O33

**Published:** 2013-06-20

**Authors:** M Jones, K Khader, B Huttner, A Huttner, C Nielson, M Rubin, M Samore

**Affiliations:** 1 Epidemiology, VA Salt Lake City HCS, Salt Lake City, USA; 2 Infection Control Program, Geneva University Hospitals and Faculty of Medicine, Geneva, Switzerland; 3 Veterans Affairs Reno Medical Center, Reno, USA

## Introduction

The prevalence of methicillin-resistant *Staphylococcus aureus* (MRSA)-carriage among hospital admissions (importation) is high in United States Veterans Affairs (VA) hospitals when compared to published studies from other settings. We investigated whether inpatient transmission alone could explain this finding.

## Methods

We used retrospective clinical data from 112 VA hospitals to calibrate a stochastic compartmental simulation model. We modeled a typical VA hospital with 70 beds and its surrounding community with 38,000 enrolled Veterans. The model consisted of 6 patient states—susceptible and colonized individuals who had never been hospitalized, were currently hospitalized, or had a history of hospitalization. Admission, length of stay, inpatient acquisition rates, and mortality rates were calibrated to observed data for MRSA-positive and negative populations. The relative rate of admission of MRSA-positive to negative populations was set to 1.2, based on the relative rate of readmission. Importation among first time admissions was 6.8%; overall it was 10.5%. Readmission rates were calibrated to reflect 15% 30 day readmission [1] . We compared a base-case scenario assuming 5% assumed MRSA prevalence among new Veterans entering the population and a scenario without inpatient transmission. Each scenario was run 200 times.

## Results

In the base-case scenario, the median importation was 10.2% (IQR 9.7-10.8%), the median outpatient prevalence was 5.0%, and the median outpatient acquisition rate was 1.5/100,000 person-years. Without inpatient transmission, the median importation dropped to 3.5% (IQR 3.3-3.6%).

## Conclusion

Dynamic theory predicts that discharge prevalence, outpatient transmission, and readmission rates influence importation. This modeling study demonstrated a large impact of inpatient transmission on MRSA importation prevalence when other factors were held constant. It appears plausible that the high importation prevalence observed in VA hospitals may be attributed to the nosocomial acquisition and readmission of prevalent patients. A sensitivity analysis is necessary to assess the robustness of this finding to our assumptions.

## Disclosure of interest

None declared.
